# Hypermethylation of the sodium channel beta subunit gene promoter is associated with colorectal cancer

**DOI:** 10.1186/s41065-024-00340-0

**Published:** 2024-10-17

**Authors:** Yabin Liu, Ya Duan, Tianliang Bai, Dexian Kong

**Affiliations:** 1 Department of General Surgery, Fourth Hospital of Hebei Medical University, Shijiazhuang, Hebei 050011 P.R. China; 2 Department of Obstetrics, Hebei General Hospital, Shijiazhuang, Hebei 050011 P.R. China; 3 Department of Gastrointestinal Surgery, Affiliated Hospital of Hebei University, Baoding, Hebei 071000 P.R. China; 4 Department of Endocrinology, Fourth Hospital of Hebei Medical University, 12 Jiankang Road, Shijiazhuang, Hebei 050011 P.R. China

**Keywords:** Colorectal cancer, SCNN1B, Methylation, Prognosis

## Abstract

**Aims:**

To better understand the role of sodium channel beta subunit (*SCNN1B*) in the initiation and progression of colorectal cancer (CRC) and to identify potential biomarkers for the early detection and prognosis of CRC.

**Methods:**

A total of 74 pairs of CRC tissues and their adjacent normal tissues were collected between October 2016 and November 2017. The methylation levels of the *SCNN1B* promoter region in CRC tissues and their adjacent normal tissues were investigated by pyrosequencing. The expression of both *SCNN1B* mRNA and protein were detected by RT‒qPCR and immunohistochemistry, respectively.

**Results:**

The results showed that the methylation levels of the *SCNN1B* promoter region were significantly higher in CRC tissues than in adjacent normal tissues. The expression levels of *SCNN1B* mRNA and protein were significantly lower in the CRC tissues than in their adjacent normal tissues. Moreover, Pearson’s correlation analysis showed that the methylation levels of the *SCNN1B* promoter were negatively correlated with the *SCNN1B* mRNA levels in CRC tissues. In addition, the high methylation levels and low mRNA expression of *SCNN1B* showed a significant association with advanced tumour stage, increased risk of lymph node metastasis and poor prognosis of CRC patients.

**Conclusion:**

This study suggested that the decreased expression of SCNN1B due to its promoter hypermethylation may play an important role in the progression and prognosis of CRC, and the methylation levels of the *SCNN1B* promoter may serve as an effective molecular marker for predicting the progression and prognosis of CRC.

**Supplementary Information:**

The online version contains supplementary material available at 10.1186/s41065-024-00340-0.

## Introduction

Colorectal cancer (CRC) is a common malignant tumour and was responsible for 940,000 mortalities worldwide in 2020 [1]. In China, the incidence and mortality of CRC have risen considerably in recent decades. Even with great advances in CRC screenings and clinical treatment, the 5-year survival rate for CRC patients remains at approximately 65% [2]. Early diagnosis is a vital strategy for decreasing CRC-associated mortality [3]. Therefore, the identification of highly sensitive and specific biomarkers that could help with early CRC detection is urgently needed.

Accumulating evidence has revealed that CRC is influenced by aberrant genetic and epigenetic alterations [4]. DNA methylation is an important epigenetic change and has been identified as a key regulator of gene activity in humans [5]. Hypermethylated promoters in tumour suppressor genes can cause gene transcriptional silencing and carcinogenesis in many human tumours, including CRC [6–8]. Deregulated DNA methylation has been found to occur early in tumorigenesis and is considered a favourable molecular marker in early cancer screening and diagnosis [9]. Previous studies have indicated several aberrantly methylated genes might function as molecular biomarkers for the early detection and prognosis of CRC [10, 11]. However, their diagnostic sensitivity and specificity remains unsatisfactory.

The *SCNN1B* gene lies on human chromosome 16p12.2 and encodes the epithelial sodium channel β-subunit (EnaC-β), which, along with other EnaC subunits, participates in maintaining sodium and water homeostasis. Beyond the physiologic regulation of the epithelial Na + channel, SCNN1B also plays a role in cellular differentiation [12, 13]. Qian et al. reported that overexpression of SCNN1B could inhibit the cell proliferation and metastasis abilities of gastric cancer cell lines [13]. Moreover, using Gene Expression Omnibus (GEO) datasets, SCNN1B expression was significantly decreased in cervical cancer, lung squamous cell carcinoma and colorectal cancer [14–16]. These data indicated that SCNN1B might be a tumour suppressor.

The methylation levels of *SCNN1B* gene promoter regions were significantly increased in gastric cancer and renal cell carcinoma [17, 18]. Moreover, hypermethylation of *SCNN1B* was a prognostic factor that predicts worse survival in patients with gastric cancer [18]. However, little is known regarding the methylation status of *SCNN1B* in CRC. In this study, we investigated the methylation levels of *SCNN1B* and its expression in CRC tissues. The aims of the study were to better understand the role of *SCNN1B* in the initiation and progression of CRC and to identify potential biomarkers for the early detection and prognosis of CRC.

## Materials and methods

### Tissue extraction

In this study, CRC tumour tissues and their corresponding adjacent normal tissues were obtained from 74 patients who underwent surgical treatment for CRC at the Fourth Hospital of Hebei Medical University from October 2016 to November 2017. All patients were confirmed to have primary CRC by postoperative histological examination without having received chemotherapy or radiotherapy prior to surgery. Normal colon tissues were obtained from ≥ 10 cm away from the CRC tumour edge. CRC tumour tissues and normal colon tissues were collected within 30 min of surgical resection and then divided into two parts: (1) one part was stored in RNAlater solution for *SCNN1B* methylation and mRNA expression analysis; (2) the other part was fixed in 4% paraformaldehyde for immunohistochemical staining. The CRC patients’ clinical and pathological data were recorded in their medical charts. Patients with any other cancers or recurrent CRC were excluded from the current study.

For the CRC patients, follow-up was performed every 3 months after surgery. Standard postoperative surveillance, including clinical symptoms, serum carcinoembryonic antigen assay, and serial computed tomography scans, was used to assess the recurrence of CRC. Disease-free survival (DFS) was defined as the time interval between surgical treatment and first tumour relapse. Overall survival (OS) was defined as the time interval between surgical treatment and death due to any cause. All participants in this study were people of Han ethnicity in northern China. This study was approved by the ethics committee of Hebei Medical University (2016KY039), and all recruited subjects provided written informed consent forms according to the Helsinki Declaration.

### DNA extraction and pyrosequencing methylation analysis

**Fig. 1:**
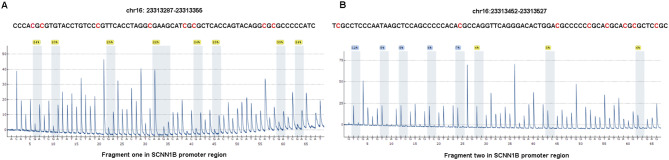
Pyrosequencing assay for detection of *SCNN1B* methylation. (**A**) Sequence of the fragment one in *SCNN1B* promoter region analyzed for methylation. Eight CpG dinucleotides are shown in red. At the bottom, a pyrogram is exhibited, visualizing the peaks of nucleotide sequence readings with integrated information about the percentage of the methylation of the investigated CpG sites. (**B**) Sequence of the fragment two in *SCNN1B* promoter region analyzed for methylation. Eight CpG dinucleotides are shown in red as well. At the bottom, a pyrogram for the CpG sites methylation pyrosequencing test

DNA was isolated from the CRC tissues and their corresponding adjacent normal tissues with the Wizard Genomic DNA Purification Kit (Promega, Madison, WI, USA) according to the manufacturer’s instructions. The purity and concentration of the extracted DNA were evaluated by a UV spectrophotometer. Bisulfite conversion was performed with the EpiTect Fast Bisulfite Conversion Kit (Qiagen, Hilden, Germany). Two fragments containing 16 CpG sites in the promoter region of *SCNN1B* were analysed in the sodium bisulfite-converted DNA (Fig. 1). The primer sequences for polymerase chain reaction (PCR) amplification and pyrosequencing, designed by the PyroMark Assay Design Software 2.0, are shown in Table 1. Pyrosequencing reactions were conducted on a PyroMark Q48 instrument with PyroMark Gold Reagents (Qiagen, Hilden, Germany). PyroQ-CpG software was used to gain the methylation percentages of each CpG site. For data analysis, the mean methylation levels of all CpG sites in each fragment were compared between the CRC tissues and their adjacent normal tissues.

**Table 1:** Primer sequences used for PCR amplification and pyrosequencing

Primers	Sequences
Fragment one	
Forward PCR Primer	5’-GTAGGGGTGTGGATGTGA-3’
Reverse PCR Primer	5’-ACCCCACCTCCCCTCAATACAT-3’
Sequencing Primer	5’-ACACTCCATCCCACC-3’
Fragment two	
Forward PCR Primer	5’-GGATGAGGGGTTTGTGGATA-3’
Reverse PCR Primer	5’-TCCCCTAACCACCTCCCT-3’
Sequencing Primer	5’-TGGGGAGGGAGAATG-3’

### RNA extraction and real-time quantitative PCR (RT‒qPCR)

Total RNA was extracted from the CRC tissues and their corresponding adjacent normal tissues with TRIzol reagent (Generay Biotech Co., Ltd., Shanghai, China). The extracted RNA was then reverse transcribed into cDNA by the Revert Aid First Strand cDNA Synthesis Kit (Thermo Scientific, MA, USA). For RT‒qPCR, the primer sequences were designed and produced by Sangon Biotech Co. Ltd. (Shanghai, China) were as follows: (a) *SCNN1B*: 5’-GTC AGC GTC TCC CTC TCC GTA G-3’ (forwards) and 5’-TCC ATC AGC TCA TCC AGG TCC TTC-3’ (reverse); and (b) *GAPDH* (used as internal control): 5’-CGG AGT CAA CGG ATT TGG TCG TA-3’ (forwards) and 5’-AGC CTT CTC CAT GGT GGT GAA GAC-3’ (reverse). The RT‒qPCR experiments were conducted on an ABI 7500 detection (Applied Biosystems, Foster City, CA, USA) system using the QuantiNova ™SYBR^®^ Green PCR Kit (Qiagen). The relative expression level of *SCNN1B* mRNA was obtained by the 2^−ΔΔCt^ method, and each reaction was carried out three times.

### Immunohistochemistry

Immunohistochemistry for SCNN1B protein was conducted as described previously [19]. Briefly, 4 μm thick sections of paraffin-embedded CRC tissues and their corresponding adjacent normal tissue samples were deparaffinized in xylene and dehydrated through descending ethanol. After removing endogenous peroxidase activity and nonspecific staining, sections were incubated with primary antibody (rabbit polyclonal anti-EnaC β antibody; Cataiog No: YT1551; ImmunoWay Biotechnology, Beijing, China; dilution 1:100) in a humidity chamber at 4 °C overnight. The following day, sections were rinsed with phosphate-buffered saline (PBS) and incubated with biotinylated secondary antibody and streptavidin-peroxidase complex. Then, the sections were rinsed in PBS, incubated with liquid DAB reagent and counterstained with haematoxylin. All sections were independently evaluated by two pathologists in a blinded manner. Immunohistochemical staining was determined according to a previously reported scoring method [20]. Scores between 3 and 6 were regarded as positive expression, and scores between 0 and 2 were regarded as negative expression.

### Statistical analysis

The statistical analysis was conducted using the SPSS software package (version 21.0; SPSS Inc., Chicago, IL). The methylation and mRNA levels of *SCNN1B* are expressed as the mean ± standard deviation. A paired-samples t test was used to evaluate the differences between CRC tissues and their corresponding adjacent normal tissues. McNemar’s test was applied to compare the difference in the frequency of positive SCNN1B expression between the two groups. Pearson’s correlation tests were used to assess the relationship between the methylation levels and mRNA expression of *SCNN1B* in CRC tissues. The associations between methylation and mRNA levels of *SCNN1B* and clinical characteristics of CRC patients were analysed by unconditional logistic regression model. The Kaplan‒Meier method with the log-rank test was applied to assess the association between methylation and mRNA levels of *SCNN1B* and the prognosis of CRC patients. Multivariate analyses were conducted with the cox proportional hazards regression model. *P* < 0.05 was considered to be significant.

## Results

### Methylation patterns of *SCNN1B* in CRC tissues and their adjacent normal tissues

**Fig. 2:**
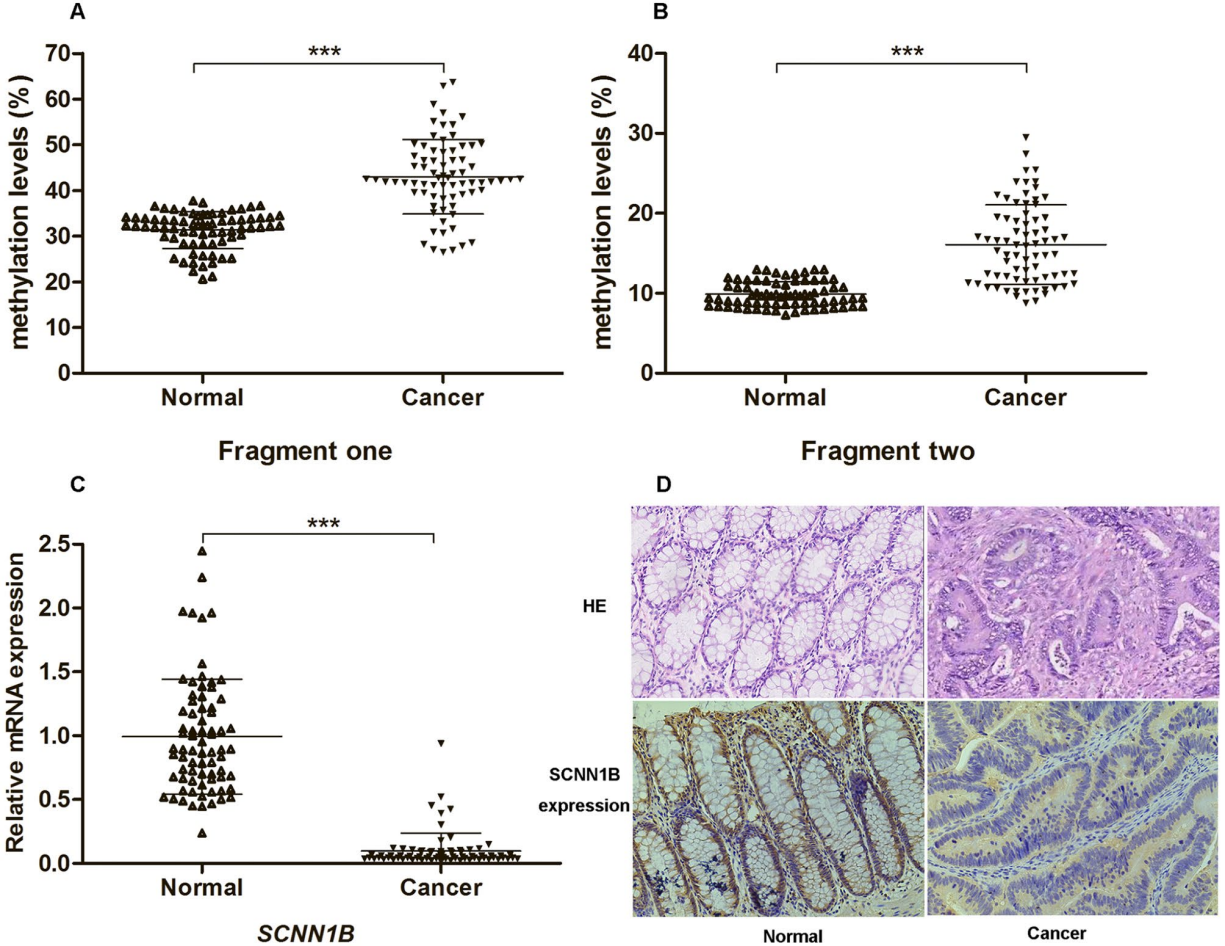
The methylation levels and expression of SCNN1B in colorectal cancer. (**A**, **B**) The mean methylation levels of fragment one and fragment two in the colorectal cancer tissues and their adjacent normal tissues. ***P < 0.001. (**C**) Relative mRNA expression of *SCNN1B* in the colorectal cancer tissues and their adjacent normal tissues. ***P < 0.001. (**D**) Representative immunohistochemical staining of SCNN1B protein in the colorectal cancer tissues and their adjacent normal tissues; Quantification of SCNN1B protein expression by scoring IHC staining in 74 paired colorectal cancer tissues and their adjacent normal tissues (*P* = 0.012)

The eight CpG sites in fragment one exhibited strong correlations among each other (all *r* > 0.522, *P* < 0.05), and similar results were found among the CpG sites of fragment two (all *r* > 0.494, *P* < 0.05). Thus, the mean methylation levels of all CpG sites in each fragment were utilized for further analysis. The results showed that the mean methylation levels of fragment one and fragment two were significantly higher in the CRC tissues than in their adjacent normal tissues (*P* < 0.001 and < 0.001, respectively) (Fig. 2A and B).

### Expression of *SCNN1B* in CRC tissues and their adjacent normal tissues

The RT‒qPCR results demonstrated that the expression levels of *SCNN1B* mRNA were significantly lower in the CRC tissues than in their adjacent normal tissues (*P* < 0.001) (Fig. 2C). To further confirm the expression of *SCNN1B* in CRC tissues, we analyzed the mRNA levels of *SCNN1B* in CRC tissues and normal colon tissues from the GEPIA2 (http://gepia2.cancer-pku.cn/) database. The database data also showed that the mRNA levels of *SCNN1B* in CRC tissues were significantly lower than those in normal colon tissues (Fig. S1). Immunohistochemical staining revealed that SCNN1B was mainly cytoplasmic in CRC and normal colorectal tissues (Fig. 2D). Compared with normal colorectal tissues, the frequency of positive SCNN1B expression was significantly decreased in CRC tissues (*P* = 0.012) (Table 2).

**Table 2:** The protein expression of SCNN1B in CRC tissues and their adjacent normal tissues

Groups	*N*	Negative expression	Positive expression	*P*
Normal tissues	74	23 (31.1%)	51 (68.9%)	
Cancer tissues	74	38 (51.4%)	36 (48.6%)	0.012

### Correlation between *SCNN1B* promoter methylation levels and its mRNA expression

Pearsonʼs correlation analysis showed that the methylation levels of the *SCNN1B* promoter were negatively correlated with the *SCNN1B* mRNA levels in CRC tissues (fragment one: *r* = − 0.562, *P* < 0.001; fragment two: *r* = − 0.430, *P* < 0.001).

### Association between methylation and mRNA levels of *SCNN1B* and the clinical characteristics of CRC patients

The clinical characteristics and some key gene variations of the CRC are summarized in Table 3. The correlation between the methylation levels of *SCNN1B* and the clinicopathological parameters of the CRC patients, such as age, sex, tumour differentiation, tumour stage, lymph node metastasis, tumour localization, RAS mutation, BRAF mutation, and MSI status, was evaluated. The median methylation levels of fragment one and fragment two of *SCNN1B* in CRC tissues were 42.4% (quartile range: 39.1-48.5%) and 15.6% (quartile range: 11.7-19.5%), respectively. Based on the median value of *SCNN1B* promoter methylation levels, the 74 CRC patients were divided into *SCNN1B* methylation^high^ (*n* = 37) and *SCNN1B* methylation^low^ (*n* = 37) groups. The results demonstrated that the methylation status of the *SCNN1B* promoter was not related to age, sex, tumour differentiation, tumour localization, RAS mutation, BRAF mutation, or MSI status in patients with CRC (Table 3). However, the high methylation levels of fragment one in the *SCNN1B* promoter showed a significant association with advanced tumour stage (*P* = 0.004) and lymph node metastasis (*P* = 0.003) in CRC patients. Moreover, the high methylation levels of fragment two were also related to an increased risk of lymph node metastasis (*P* = 0.015).

**Table 3:** Association of *SCNN1B* methylation levels and clinical characteristics of CRC patients by logistic regression

Clinical characteristics	*n*	Methylation^high^ of fragment one			Methylation^high^ of fragment two		
		Cases (%)	Odds ratio (OR)	*P*	Cases (%)	Odds ratio (OR)	*P*
Age (years)							
< 60	33	18 (54.5)	Reference		17 (51.5)	Reference	
≥ 60	41	19 (46.3)	0.72 (0.29–1.81)	0.483	20 (48.8)	0.90 (0.36–2.24)	0.815
Gender							
Male	41	21 (51.2)	Reference		19 (46.3)	Reference	
Female	33	16 (48.5)	0.90 (0.36–2.24)	0.815	18 (54.5)	1.39 (0.55–3.49)	0.483
Differentiation							
Level I-II	54	26 (48.1)	Reference		28 (51.9)	Reference	
Level III	20	11 (55.0)	1.32 (0.47–3.69)	0.601	9 (45.0)	0.76 (0.27–2.13)	0.601
TNM stage							
I-II	46	17 (37.0)	Reference		19 (41.3)	Reference	
III-IV	28	20 (71.4)	4.26 (1.55–11.77)	0.004	18 (64.3)	2.56 (0.97–6.75)	0.055
Lymphatic metastasis							
No	48	18 (37.5)	Reference		19 (39.6)	Reference	
Yes	26	19 (73.1)	4.52 (1.59–12.87)	0.003	18 (69.2)	3.43 (1.25–9.47)	0.015
Tumor location							
Colon	28	16 (57.1)	Reference		15 (53.6)	Reference	
Rectal	46	21 (45.7)	0.63 (0.24–1.62)	0.338	22 (47.8)	0.79 (0.31–2.04)	0.632
RAS mutation							
(-)	33	16 (48.5)	Reference		15 (45.5)	Reference	
(+)	41	21 (51.2)	1.12 (0.45–2.79)	0.815	22 (53.7)	1.39 (0.55–3.49)	0.483
BRAF mutation							
(-)	65	33 (50.8)	Reference		31 (47.7)	Reference	
(+)	9	4 (44.4)	0.78 (0.19–3.15)	0.722	6 (66.7)	2.19 (0.51–9.53)	0.286
MSI Status							
MSS	67	33 (49.3)	Reference		35 (52.2)	Reference	
MSI-high	7	4 (57.1)	1.37 (0.29–6.61)	0.691	2 (28.6)	0.37 (0.07–2.02)	0.233

Abbreviations MSI: microsatellite instability; MSS: microsatellite stable

The median mRNA level of *SCNN1B* in CRC tissues was 0.054 (quartile range: 0.038–0.093). Based on the median value of *SCNN1B* mRNA levels, the 74 CRC patients were divided into *SCNN1B* expression^high^ (*n* = 37) and *SCNN1B* expression^low^ (*n* = 37) groups. The expression level of *SCNN1B* was not associated with age, sex, tumour differentiation, tumour localization, RAS mutation, BRAF mutation, or MSI status in patients with CRC (Table 4). However, the low mRNA levels of *SCNN1B* had a significant relationship with advanced tumour stage (*P* = 0.016) and lymph node metastasis (*P* = 0.014) in CRC patients (Table 4).

**Table 4:** Association of *SCNN1B* mRNA levels and clinical characteristics of CRC patients by logistic regression

Clinical characteristics	*n*	SCNN1B mRNA levels		Odds ratio (OR)	*P*
		Low (%)	High (%)		
Age (years)					
< 60	33	19 (57.6)	14 (42.4)	Reference	
≥ 60	41	18 (43.9)	23 (56.1)	0.58 (0.23–1.46)	0.242
Gender					
Male	41	23 (56.1)	18 (43.9)	Reference	
Female	33	14 (42.4)	19 (57.6)	0.58 (0.23–1.46)	0.242
Differentiation					
Level I-II	54	28 (51.9)	26 (48.1)	Reference	
Level III	20	9 (45.0)	11 (55.0)	0.76 (0.27–2.13)	0.601
TNM stage					
I-II	46	18 (39.1)	28 (60.9)	Reference	
III-IV	28	19 (67.9)	9 (32.1)	3.28 (1.22–8.84)	0.016
Lymphatic metastasis					
No	48	19 (39.6)	29 (60.4)	Reference	
Yes	26	18 (69.2)	8 (30.8)	3.43 (1.25–9.47)	0.014
Tumor location					
Colon	28	15 (53.6)	13 (46.4)	Reference	
Rectal	46	22 (47.8)	24 (52.2)	0.79 (0.31–2.04)	0.632
RAS mutation					
(-)	33	17 (51.5)	16 (48.5)	Reference	
(+)	41	20 (48.8)	21 (51.2)	0.90 (0.36–2.24)	0.815
BRAF mutation					
(-)	65	32 (49.2)	33 (50.8)		
(+)	9	5 (55.6)	4 (44.4)	1.29 (0.32–5.24)	0.722
MSI Status					
MSS	67	33 (49.3)	34 (50.7)	Reference	
MSI-high	7	4 (57.1)	3 (42.9)	1.37 (0.29–6.61)	0.691

Abbreviations MSI: microsatellite instability; MSS: microsatellite stable

### Association between methylation and mRNA levels of *SCNN1B* and the prognosis of CRC patients

**Fig. 3:**
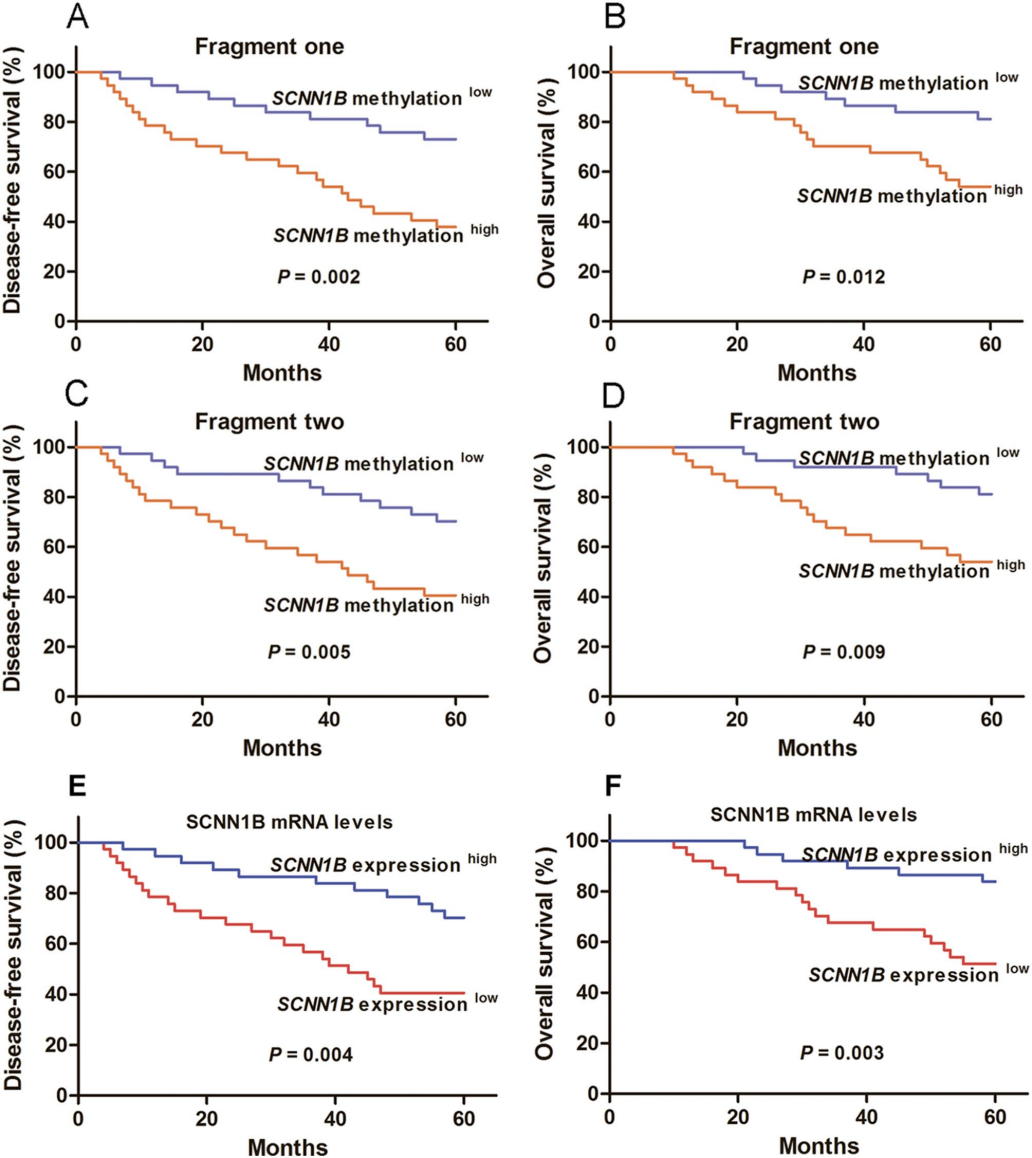
Kaplan–Meier analysis estimates the prognosis of colorectal cancer patients based on the methylation levels and mRNA expression of the *SCNN1B*. (**A**) Mean DFS of colorectal cancer patients in the *SCNN1B* methylation^low^ group and *SCNN1B* methylation^high^ group of fragment one were 51.8 and 38.6 months, respectively (*P* = 0.002). (**B**) Mean OS of colorectal cancer patients in the *SCNN1B* methylation^low^ group and *SCNN1B* methylation^high^ group of fragment one were 55.3 and 46.9 months, respectively (*P* = 0.012). (**C**) Mean DFS of colorectal cancer patients in the *SCNN1B* methylation^low^ group and *SCNN1B* methylation^high^ group of fragment two were 51.9 and 38.5 months, respectively (*P* = 0.005). (**D**) Mean OS of colorectal cancer patients in the *SCNN1B* methylation^low^ group and *SCNN1B* methylation^high^ group of fragment two were 56.2 and 46.1 months, respectively (*P* = 0.009). (**E**) Mean DFS of colorectal cancer patients in the *SCNN1B* expression^low^ group and *SCNN1B* expression^high^ group were 38.2 and 52.3 months, respectively (*P* = 0.004). (**F**) Mean OS of colorectal cancer patients in the *SCNN1B* expression^low^ group and *SCNN1B* expression^high^ group were 46.2 and 56.0 months, respectively (*P* = 0.003)

Kaplan‒Meier analysis showed that the methylation levels and mRNA expression of *SCNN1B* were associated with the DFS and OS of CRC patients (Fig. 3). The CRC patients in the *SCNN1B* methylation^high^ group of fragment one had significantly shorter DFS and OS times than those in the *SCNN1B* methylation^low^ group (*P* = 0.002 and 0.012, respectively). Similar results were observed in the *SCNN1B* methylation^high^ group of fragment two (*P* = 0.005 and 0.009, respectively). Furthermore, patients in *SCNN1B* expression^low^ group exhibited a shorter DFS and OS times than those in the *SCNN1B* expression^high^ group (*P* = 0.004 and 0.003, respectively). Survival analysis of an independent CRC cohort (GSE17538) also revealed that low mRNA expression of *SCNN1B* was associated with worse survival in CRC patients (Fig. S2). The significant association between methylation and mRNA levels of *SCNN1B* and the CRC patients’ prognosis was maintained by multivariate analysis containing tumour stage and lymph node metastasis (Table 5).

**Table 5:** Association between methylation and mRNA levels of *SCNN1B* and 5-year clinical outcomes of colorectal cancer patients by multivariate cox regression model

Variables	No. of recurrence (%)	Disease-free survival		No. of death (%)	Overall survival	
		HR (95% CI) ^a^	*P* ^a^		HR (95% CI) ^a^	*P* ^a^
Fragment one						
Methylation^low^	10 (27.0)	reference		7 (18.9)	reference	
Methylation^high^	23 (62.2)	2.44 (1.13–5.26)	0.023	17 (45.9)	2.04 (0.82–5.09)	0.125
Fragment two						
Methylation^low^	11 (29.7)	reference		7 (18.9)	reference	
Methylation^high^	22 (59.5)	2.61 (1.22–5.56)	0.013	17 (45.9)	2.83 (1.10–7.25)	0.031
*SCNN1B* mRNA						
Expression^low^	22 (59.5)	reference		18 (48.6)	reference	
Expression^high^	11 (29.7)	0.40 (0.19–0.84)	0.015	6 (16.2)	0.33 (0.13–0.84)	0.020

Abbreviations HR: Hazard ratio; CI: Confidence interval

^a^Adjusted for tumour stage and lymph node metastasis

## Discussion

SCNN1B is a methylation-related suppressor gene mentioned in renal cell carcinoma and gastric cancer [17, 18]. The anti-tumor effect of SCNN1B is mediated via degradation of the endoplasmic reticulum chaperone GRP78, which is frequently up-regulated during cancer progression to counter unfolded protein response (UPR), maintain ER homeostasis and promote cell survival [13]. Hypermethylation of SCNN1B was identified as an prognostic factor in gastric cancer patients [18]. In this study, we assessed the association between the methylation status of the *SCNN1B* promoter region and CRC. The results showed that *SCNN1B* was significantly hypermethylated in tumour tissues of CRC patients compared to their adjacent normal tissues. Furthermore, high methylation levels of the *SCNN1B* promoter were correlated with advanced tumour stage, an increased risk of lymph node metastasis and poor prognosis in CRC patients. To our knowledge, this study is the first to explore the role of the *SCNN1B* methylation profile in the initiation and progression of CRC.

Previous studies have reported that the methylation patterns of several genes, such as *TSHZ3*, *SM22α*, *RASGRF1* and *OGDHL*, were altered in CRC [21–24]. Global methylation analysis also implied that significant methylation differences existed in CRC tissues compared to normal colorectal tissues [25, 26]. In the present study, we found that the promoter region of *SCNN1B* was significantly hypermethylated in CRC tissues, and the mRNA and protein expression levels of SCNN1B were lower in CRC tissues than in their adjacent normal tissues. Moreover, the methylation levels of the *SCNN1B* promoter were negatively correlated with *SCNN1B* mRNA expression in CRC tissues. Our findings were similar to the results of Qian et al. [13], who reported that the expression of *SCNN1B* mRNA is silenced by its promoter methylation in gastric cancer tumour tissues, and SCNN1B overexpression could inhibit multiple features of cancer cell pathophysiology in vitro and in vivo. Our results suggested that the hypermethylated promoter region of *SCNN1B* may be involved in the pathogenesis of CRC by silencing the *SCNN1B* gene.

Aberrant DNA methylation profiles have been shown to be significantly associated with cancer progression and prognosis [27–29]. Peng et al. [18] reported that hypermethylation of *SCNN1B* is an independent prognostic factor for poor survival in patients with gastric cancer. In this study, we also investigated the correlation between the methylation levels of the *SCNN1B* promoter and the clinicopathologic features and prognosis of CRC patients. Our results showed that elevated *SCNN1B* methylation levels in CRC tissues were significantly related to advanced tumour stage and a high risk of lymph node metastasis. CRC patients with hypermethylated *SCNN1B* promoter had poorer clinical outcomes than those with hypomethylated *SCNN1B* promoter. These data indicate that the methylation status of the *SCNN1B* promoter may serve as a potential survival marker for CRC.

There are several limitations in this study. First, the sample size of this study is relatively small. Thus, a large cohort is needed to confirm the clinical significance of hypermethylated *SCNN1B* promoters in CRC patients. Second, blood-based tests are less invasive and more convenient than tumour tissue biopsy. However, the current study only detected the methylation levels of the *SCNN1B* promoter in CRC tissues. Finally, we did not evaluate the methylation levels and expression of SCNN1B in the control group with benign colorectal polyps or adenomas.

In conclusion, our study suggested that the decreased expression of SCNN1B due to hypermethylation of its promoter may play an important role in the progression and prognosis of CRC. Thus, the methylation levels of the *SCNN1B* promoter may serve as an effective molecular marker for predicting the progression and prognosis of CRC.

## Electronic supplementary material

Below is the link to the electronic supplementary material.

Supplementary Material 1: Supplementary Fig. 1 The mRNA levels of *SCNN1B* in colorectal cancer tissues from the GEPIA2 database. A total of 275 colorectal cancer tissues and 349 normal colon tissues. Red color = Tumor tissues; Black color = Normal colon tissues. *P < 0. 01. COAD: Colon adenocarcinoma

Supplementary Material 2: Supplementary Fig. 2 Low mRNA levels of *SCN*N1B predicts poor prognosis in colorectal cancer patients (GSE17538 cohort)

## Data Availability

No datasets were generated or analysed during the current study.
