# Supplementary figures and images for: Hypermethylation of the sodium channel beta subunit gene promoter is associated with colorectal cancer
Source: Hereditas. 2024 Oct 17;161:39. doi: 10.1186/s41065-024-00340-0 (PMC11484387; doi:10.1186/s41065-024-00340-0)

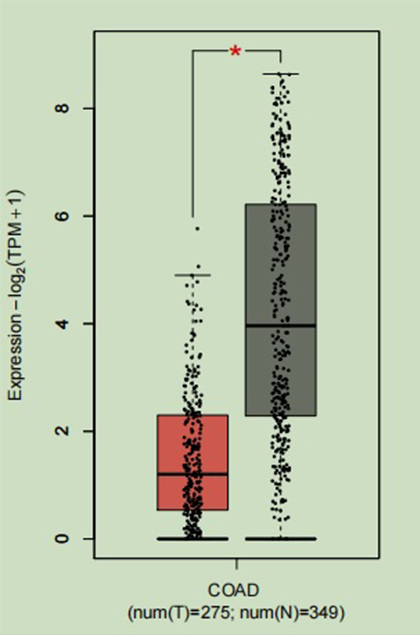

Supplement: Supplementary file 1 — Supplementary Material 1: Supplementary Fig. 1 The mRNA levels of SCNN1B in colorectal cancer tissues from the GEPIA2 database. A total of 275 colorectal cancer tissues and 349 normal colon tissues. Red color = Tumor tissues; Black color = Normal colon tissues. *P < 0. 01. COAD: Colon adenocarcinoma [file 41065_2024_340_MOESM1_ESM.tif]

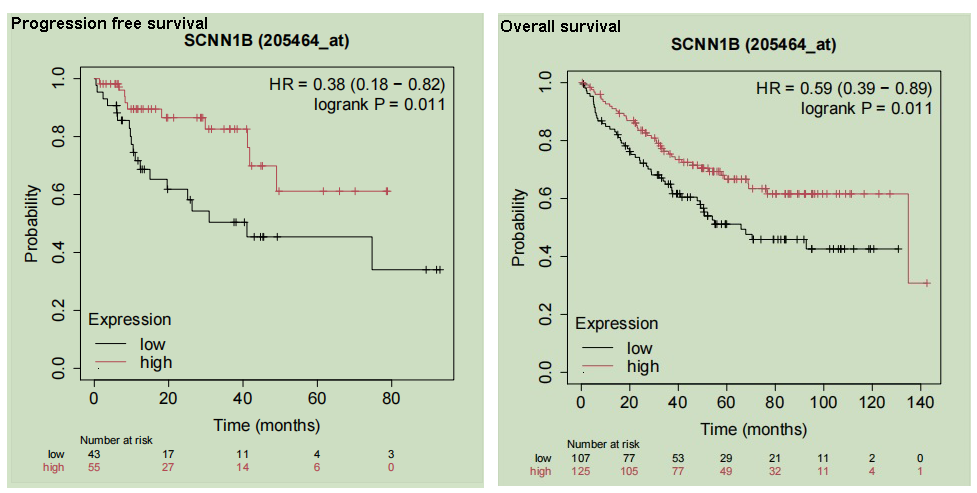

Supplement: Supplementary file 2 — Supplementary Material 2: Supplementary Fig. 2 Low mRNA levels of SCNN1B predicts poor prognosis in colorectal cancer patients (GSE17538 cohort) [file 41065_2024_340_MOESM2_ESM.tif]
